# Eustachian Valve Endocarditis in a Patient With Charcot-Marie-Tooth Disease

**DOI:** 10.7759/cureus.37375

**Published:** 2023-04-10

**Authors:** Ross Moyer, Farhan Azad, Zachary Brumberger

**Affiliations:** 1 Internal Medicine, University at Buffalo, Buffalo, USA; 2 Cardiology, University at Buffalo, Buffalo, USA

**Keywords:** trans-esophageal echocardiogram, echo, right infectious endocarditis, staph aureus bacteremia, eustachian valve endocarditis

## Abstract

Endocarditis is a serious infectious disease of the endocardial surface of the heart, predominantly involving the heart valves, and it results from the colonization and proliferation of microorganisms within the bloodstream. The condition primarily affects individuals with underlying cardiac abnormalities or those who have undergone invasive procedures. Symptoms may include pyrexia, fatigue, arthralgia, and new cardiac murmur. We present a case of a young male patient who had recently undergone surgery and developed eustachian valve endocarditis (EVE), a condition scarcely described in the literature.

## Introduction

Eustachian valve endocarditis (EVE) is a rare but potentially fatal infection of the eustachian valve, an embryological remnant of the inferior vena cava valve, within the right atrium of the heart. This typically occurs through seeding from bacteremia with an initial distant focus of infection or a recent invasive procedure [1]. Due to its rarity and diverse clinical presentation, the diagnosis and management of EVE can be challenging. In this report, we describe a case of EVE in a patient with no previous cardiac history who presented with fever and chills after an operation. We highlight the importance of early recognition and appropriate management of this unusual but potentially life-threatening condition.

## Case presentation

A 35-year-old male with a history of Charcot-Marie-Tooth (CMT) disease presented with complaints of fever and chills. He had recently undergone total left knee arthroplasty (TKA) and noticed two days of worsening erythema and drainage around the surgical site. He denied any knee pain, chest pain, shortness of breath, cough, or intravenous (IV) drug, or alcohol use. Vital signs revealed a temperature of 37.4 °C, a blood pressure of 103/63 mmHg, and a heart rate of 87 beats per minute. His physical exam was remarkable for severe erythema extending from his left knee to his left foot with numerous open sores. The patient's laboratory investigations are shown in Table 1.

**Table 1:** Laboratory investigations BUN: blood urea nitrogen; ESR: erythrocyte sedimentation rate; CRP: c-reactive protein

Investigations	Results	Reference range
Complete blood count		
White blood cells	12.9 x 10^9^/L	4.0–10.5 x 10^9^/L
Hemoglobin	9.2 g/dL	12.0–16.0 g/dL
Platelet count	296 x 10^9^/L	140–400 x 10^9^/L
Basic metabolic panel		
Sodium level	138 mmol/L	133–147 mmol/L
Potassium level	3.7 mmol/L	3.5–5.6 mmol/L
Chloride	107 mmol/L	96–110 mmol/L
Carbon dioxide	11 mmol/L	20–32 mmol/L
Anion gap	20 mmol/L	5–15 mmol/L
BUN	113 mg/dL	5–27 mg/dL
Creatinine	3.85 mg/dL	0.40–1.40 mg/dL
Glucose level	93 mg/dL	60–100 mg/dL
Venous blood gas		
pH	7.30	7.35–7.45
CO_2_	28 mmHg	35–45 mmHg
Other laboratory values		
Lactate	1.1 mmol/L	0.5–2.0 mmol/L
ESR	125 mm/hr	0.0–12.0 mm/hr
CRP	466 mg/L	0.2–10.0 mg/L

**Figure 1:**
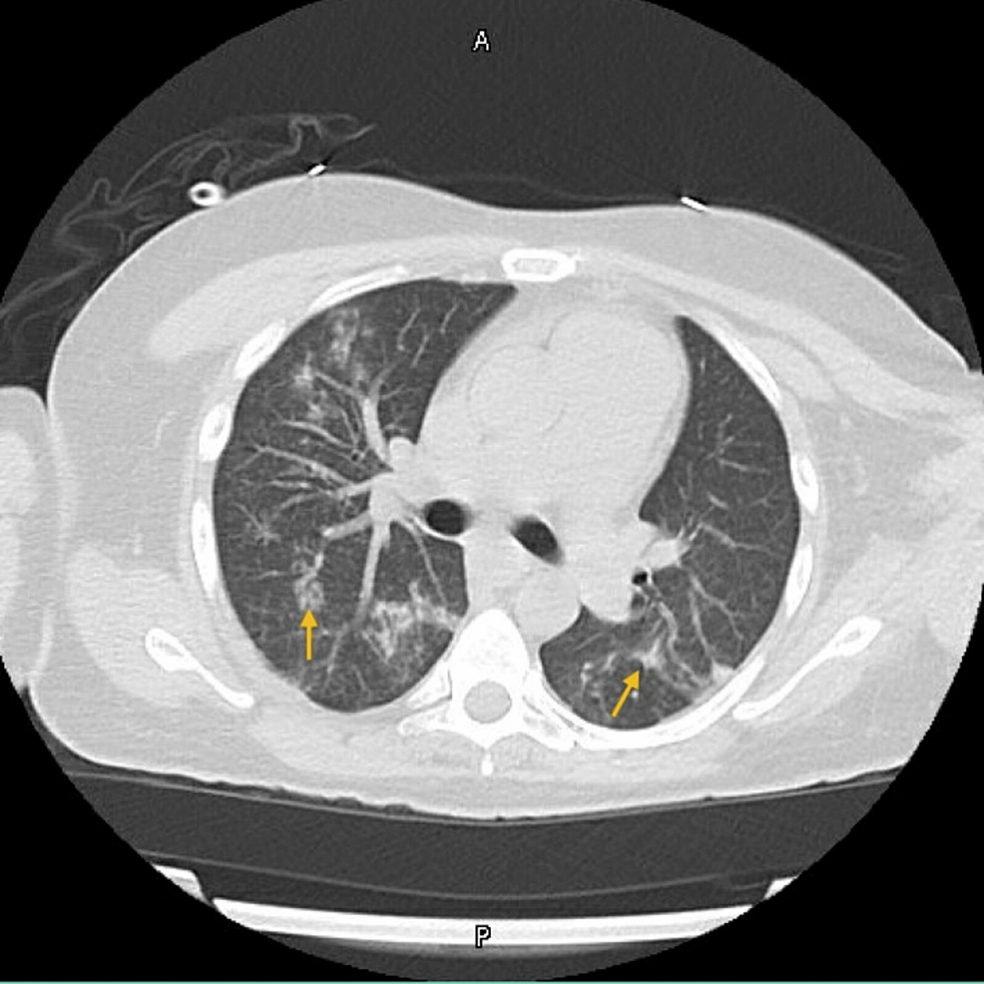
CT chest showing nodular infiltrates concerning for septic emboli (arrows) CT: computed tomography

CT with contrast of the left knee revealed a large fluid collection along the anterior aspect of the left lower extremity, with generalized edema suspicious for cellulitis. Chest CT revealed multiple nodular lung infiltrates, suspicious for pulmonary septic emboli (Figure 1).

**Figure 2:**
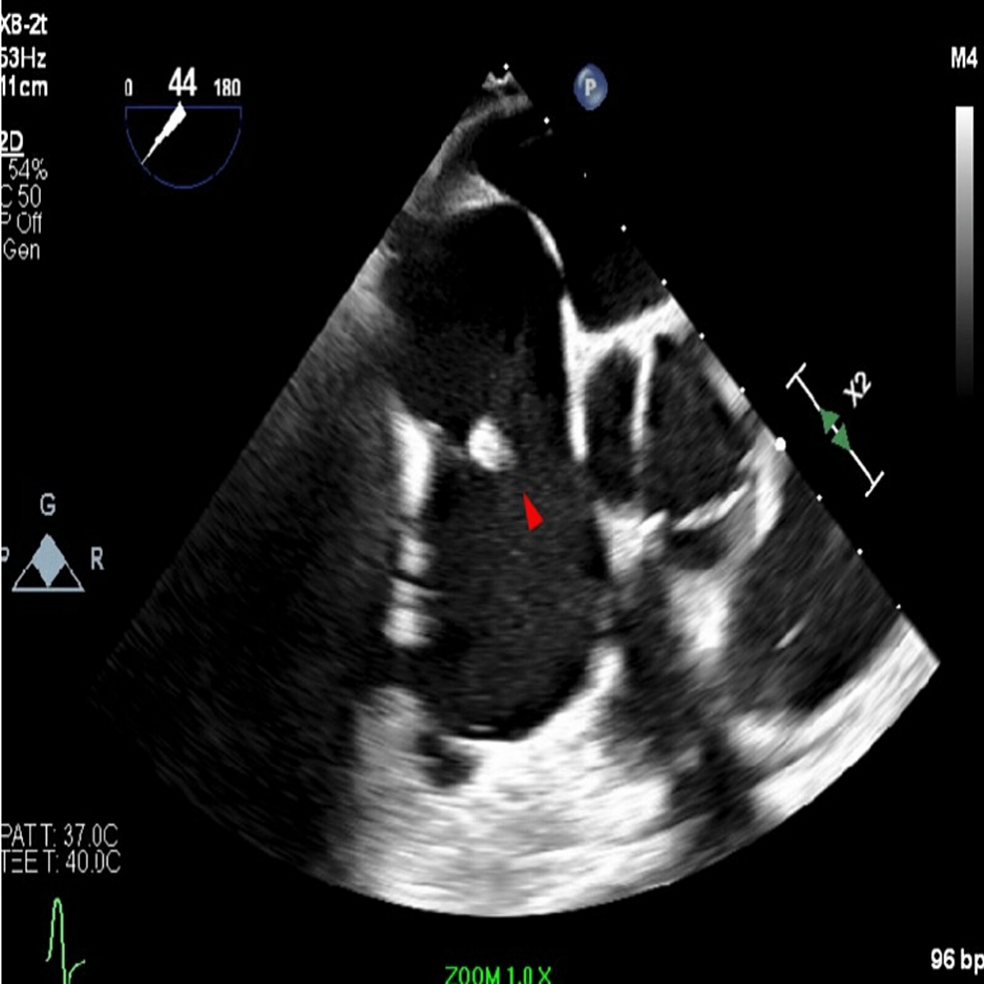
TTE showing a mobile right atrial mass attached to the valve TTE: transthoracic echocardiogram

The patient underwent an urgent incision and drainage of the left knee, resulting in the aspiration of 20 mL of dark brown purulent fluid. Blood and joint aspirate cultures grew moderate *Staphylococcus aureus* (*S. aureus*) sensitive to methicillin (MSSA). Joint fluid culture revealed a white blood cell count of 95,000/mm^3^ (reference range: 13-180 mm^3^) and it was negative for crystals. The patient was given IV fluids, and nafcillin and admitted to the ICU due to MSSA bacteremia secondary to left knee septic arthritis and left lower extremity cellulitis. The patient had persistent bacteremia based on blood cultures and showed signs of shock including hypotension and decreased urinary output. Further investigation with a transthoracic echocardiogram (TTE) uncovered a mobile right atrial mass (Figure 2). 

**Figure 3:**
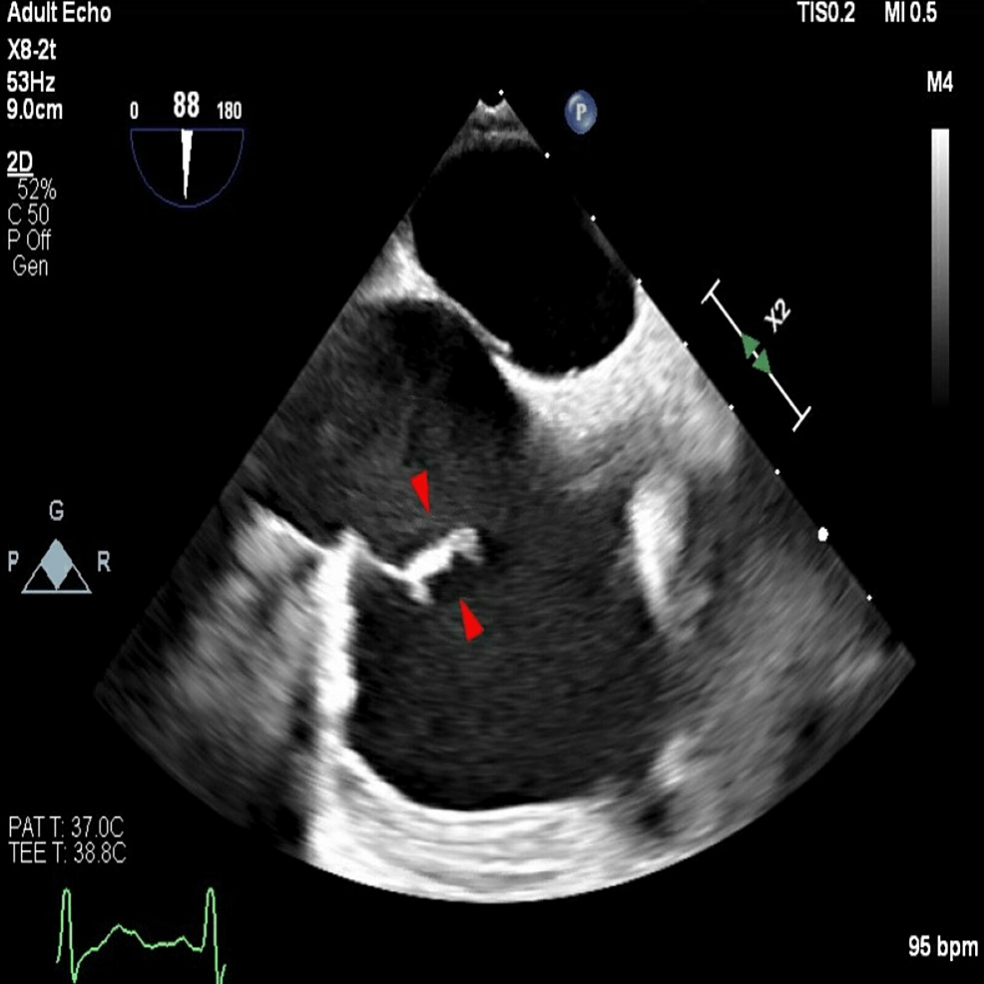
TEE showing a thickened eustachian valve within the right atrium visible with apparent fibrinous aggregate or vegetation, suspicious for endocarditis TEE: transesophageal echocardiogram

TTE was followed by a transesophageal echocardiogram (TEE), which revealed an unusually thickened eustachian valve within the right atrium visible with apparent fibrinous aggregate or vegetation, suspicious for endocarditis (Figure 3).

The patient was deemed not a candidate for percutaneous aspiration of the vegetation. He improved hemodynamically and was transferred from the ICU to the medical rehabilitation unit where he completed a six-week course of nafcillin. He was scheduled for an outpatient follow-up TEE.

## Discussion

EVE is a rare condition that is unfortunately not well-documented in the literature. The incidence of EVE is estimated to be around 3% in right-sided infective endocarditis [2]. However, the condition is associated with significant morbidity and mortality. Early diagnosis and appropriate management are crucial for a good outcome. In fact, it has been reported that embolic events and in-hospital death occur in roughly 21% and 17% of cases respectively [3]. Patient populations that are at increased risk include IV drug users and those with previously implanted hardware. In addition, uncontrolled diabetes is a serious risk factor and is also associated with poor prognostic outcomes [4]. In our patient, the risk of infection was heightened as CMT can cause profound sensory loss leading to severe and difficult-to-treat infections [5].

*S. aureus* is the usual culprit in EVE, accounting for roughly 50% of the cases, although there have been reports of Gram-negative bacteria also playing a role in the pathology [6]. Diagnosis of EVE can be challenging due to its rarity and nonspecific symptoms. Echocardiography is a valuable diagnostic tool, as it can visualize the eustachian valve and detect any vegetations or masses on the valve, which are suggestive of infection. Recent studies have shown that TEE is a more sensitive modality for right-sided endocarditis than TTE, being able to detect smaller vegetations and complications such as valve perforations [7]. Our patient underwent both TTE and TEE, and the diagnosis was made based on TEE findings.

Treatment of EVE typically involves a combination of targeted antibiotic therapy and surgical intervention, which may include valve replacement or repair. While surgical intervention for vegetation removal is also an option, it requires the patient to be temporarily put on cardiopulmonary bypass [8]. Percutaneous intervention is evolving as a new treatment option for these difficult cases. This method carries less risk as the cardiopulmonary bypass is not required and has been shown to reduce the size of the vegetation by as much as 60% [9]. However, percutaneous aspiration could also cause displacement of the vegetation into the pulmonary circulation, causing distal emboli and bleeding-related complications [10]. Unfortunately, our patient did not qualify for either of these therapies due to his concurrent infections and comorbidities. He showed improvement on antibiotics and was eventually discharged.

## Conclusions

EVE is a rare but serious condition that can lead to significant morbidity and mortality. Clinicians should maintain a high index of suspicion for EVE in patients with a history and findings of infective endocarditis, especially in those with nonspecific symptoms. It is an often-missed diagnosis. Early diagnosis and appropriate management are essential for a favorable outcome. Multidisciplinary care, including consultation with infectious disease specialists for recommendations on antibiotics and physiatry for rehabilitation, might be needed in patients requiring a prolonged hospital stay or those with extensive disease.
